# A Bayesian reevaluation of randomized controlled trials in assisted reproductive technology: quantifying evidence strength for null and alternative hypotheses

**DOI:** 10.3389/fendo.2026.1860725

**Published:** 2026-06-12

**Authors:** Jiayi Gao, Tian Tian, Kalbinur Kayimu, Yi Yuan, Yongyan Chen, Yu Fu, Rui Chen, Fang Liu, Yunjun Zhang, Yuanyuan Wang

**Affiliations:** 1 State Key Laboratory of Female Fertility Promotion, Center for Reproductive Medicine, Department of Obstetrics and Gynecology, Peking University Third Hospital; National Clinical Research Center for Obstetrics and Gynecology (Peking University Third Hospital); Key Laboratory of Assisted Reproduction (Peking University), Ministry of Education; Beijing Key Laboratory of Collaborative Innovation in Frontier Technologies for Population Quality, Beijing, China; 2 Department of Biostatistics, School of Public Health, Peking University Health Science Center, Beijing, China

**Keywords:** Bayes Theorem, randomized controlled trials as topic, negative results, reproductive techniques, assisted, secondary data analysis

## Abstract

**Introduction:**

Many randomized controlled trials (RCTs) in assisted reproductive technology (ART) face widespread misinterpretation of statistically non-significant results within the conventional frequentist framework. This study aimed to reevaluate high-quality ART RCTs using Bayesian methods to quantify the strength of the evidence for both the null and alternative hypotheses.

**Methods:**

We systematically searched ART-related RCTs published in JAMA, The Lancet, The BMJ, and NEJM between 2010 and 2025. A total of 35 trials were ultimately included for Bayesian reanalysis, which used Bayes factors (BF₁₀) to evaluate the primary outcomes. Sensitivity analyses were performed to confirm the robustness of our primary findings.

**Results:**

The Bayesian and frequentist results were consistent in 85.7% of the studies. Among these consistent results, 62.9% supported the null hypothesis and 22.9% supported the alternative hypothesis. In 11.4% of the studies, non-significant P-values were paired with inconclusive Bayes factors, indicating data insensitivity. Another 2.9% showed significant P-values but inconclusive BF10. Overall, 71.4% of studies reported non-significant primary outcomes, with an increasing trend observed over time.

**Conclusion:**

Bayesian analysis offers a useful framework for interpreting "negative results" in ART RCTs. It effectively complements traditional statistics to improve the interpretation of evidence and inform future trial design in ART.

## 1 Introduction

In recent years, the incidence of infertility has increased significantly. According to the World Health Organization (WHO), approximately 15% of couples of reproductive age worldwide face infertility issues, with even higher rates in some regions (1). Assisted Reproductive Technology (ART) is a pivotal treatment that mainly helps patients achieve pregnancy by manipulating gametes and embryos. Since 1978, ART has evolved continuously and has been widely used clinically. Global data from the International Monitoring Committee on Assisted Reproductive Technology (ICMART) indicate that approximately 3.1 million ART cycles were performed in 2016, resulting in more than 723,000 live births, with the number continuing to grow annually (2, 3).

ART has evolved through three generations: the first generation of *in vitro* fertilization-embryo transfer (IVF-ET), the second generation of intracytoplasmic sperm injection (ICSI), and the third generation of preimplantation genetic diagnosis/screening (PGD/PGS). These advances have enabled not only the treatment of diverse infertility causes but also the prevention of genetic disease transmission (4, 5). However, fertility treatment is a complex process involving maternal, procedural, and cellular levels. In particular, a growing number of studies suggest that ART may affect early embryonic development, which may lead to birth defects or long-term health risks (6). According to the Developmental Origins of Health and Disease (DOHaD) theory, early-life environment may influence disease susceptibility and phenotypic expression in adulthood (7). It is hypothesized that the artificial procedures used in ART, such as maternal hormone therapy and gamete and embryo manipulation, may induce disease risk in offspring (8, 9).

Therefore, the choice of the most appropriate ART approach according to individual conditions remains controversial in clinical practice. For instance, while IVF-ET is suitable for various causes of infertility, ICSI significantly improves fertilization and pregnancy rates in cases of severe male factor infertility. However, among infertile couples with non-severe male factors, studies have shown that ICSI offers no significant advantage over IVF-ET (10). The lack of a gold standard in ART underscores the urgent need for more robust evidence from high-quality clinical trials. Randomized controlled trials (RCTs) provide the highest level of evidence for guiding treatment decisions. However, the number of registered RCTs in reproductive medicine is very low. For instance, the ClinicalTrials.gov database contains more than 60,000 registered trials for cardiovascular diseases, compared to only approximately 1,200 for reproductive medicine. In addition, a large proportion of studies report statistically non-significant (“negative”) results and a lack of clear clinical guidance. This situation highlights a significant deficit in contemporary reproductive clinical research (11).

The vast majority of RCTs adopt a frequentist statistical framework, which relies heavily on the P-value. The classic definition of the P-value is the probability of observing the current data under the assumption that the null hypothesis (H_0_) is true. In practice, researchers often misinterpret “P > 0.05” as “evidence of no effect,” thereby confusing two fundamentally different concepts: “absence of evidence” and “evidence of absence” (12). The former may merely reflect an insufficient sample size or high variability, while the latter indicates that the data genuinely support H_0_. This misunderstanding is largely due to a limitation of the P-value: it cannot quantify the strength of evidence in favor of H_0_ or the alternative hypothesis (H_1_). In contrast, Bayesian methods evaluate research hypotheses based on posterior probability and provide a more detailed interpretation of negative results by distinguishing “no difference” from “insufficient data” (13).

Awareness of these limitations has prompted Bayesian reanalysis of published RCTs across diverse medical fields. For instance, in critical care medicine, Yarnell et al. demonstrated that Bayesian analysis could identify potential intervention benefits even when results were non-significant (14). Similarly, systematic reanalyses in gerontological psychology and plastic surgery have successfully distinguished “negative” results that nevertheless show substantial evidence for H_0_ from those that are truly inconclusive (15, 16).

A systematic Bayesian reevaluation of high-quality RCTs in ART has not been conducted yet. Therefore, this study aims to reevaluate high-quality research in the field of reproductive medicine using a Bayesian method, with particular attention to quantifying the strength of evidence and examining factors associated with negative results, thereby providing insights for clinical decision-making and future research directions.

## 2 Materials and methods

### 2.1 Literature search and data collation

We systematically searched for RCTs published between 1 January 2010 and 31 December 2025, in four high-impact medical journals: JAMA, The Lancet, The BMJ, and NEJM. These journals were selected for their clinical relevance and methodological rigor in trial design. Eligible trials evaluated an ART intervention in infertile couples and reported a primary clinical outcome with complete data for reanalysis. After removing duplicates, two reviewers independently screened titles and abstracts, and 41 potentially eligible articles were identified for full-text review. Studies were excluded if they were not RCTs, did not primarily evaluate an ART intervention, or had incomplete outcome reporting. Ultimately, 35 RCTs met all inclusion criteria and were analyzed within a Bayesian framework.

The literature search was conducted on 31 December 2025, with the aim of including all RCTs indexed in PubMed by the time the study was initiated. The complete search strategy and flow diagram are detailed in the **Supplementary Document**. The full information for all 35 included RCTs are provided in **Supplementary Table 1**, and are cited as references (10, 17–50).

### 2.2 Data extraction

Among the 35 included RCTs, we extracted the following information: study identification (title, first author, region, and publication year), design (e.g., superiority and number of arms), sample size, intervention and comparator details, primary outcome, and statistical results, including event counts, P-values, risk ratios (RRs), incidence rate ratios (IRRs), odds ratios (ORs), and 95% confidence intervals (CIs). The detailed characteristics and results of the included studies are provided in **Supplementary Tables 1**, **2**.

To minimize selection bias, we preferentially extracted results from the intention-to-treat (ITT) population when both ITT and per-protocol analyses were reported. For trials with multiple intervention arms or factorial designs, only the comparison relevant to the primary research question was retained. All steps were performed manually by the reviewers.

The methodological quality and risk of bias of each included RCT were assessed using the revised Cochrane Risk of Bias (RoB 2) tool for randomized trials. The results of this assessment are summarized in **Supplementary Table 3**. The primary objective of this study was a methodological reevaluation of individual trials, rather than providing a clinical recommendation for a specific intervention. Therefore, formal grading of the overall certainty of the body of evidence was not performed.

### 2.3 Bayes reanalysis framework

**Figure 1:**
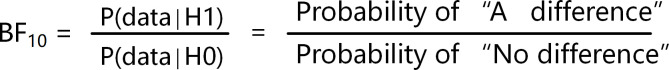
Definition of BF**_10_**.

We employed the Bayes Factor (BF_10_) to quantify the strength of evidence for H_1_ relative to H_0_ (51). The definition and calculation principle of BF_10_ are visually presented in **Figure 1**. BF_10_ is defined as the ratio of the marginal likelihood of the data under H_1_ to that under H_0_.

The following criteria were adopted for evidence interpretation (52, 53): “BF_10_ > 10” provides strong evidence in support of H_1_; “3 < BF_10_ < 10” indicates moderate evidence for H_1_; “1/3 < BF_10_ < 3” indicates inconclusive evidence; and “BF_10_ < 1/3” provides evidence in support of H_0_. For example, a BF_10_ value of 5 indicates that the observed data are five times more probable under H_1_ than under H_0_. Unlike the P-value, BF_10_ directly compares the rationality of the hypotheses based on the actual data, without relying on the framework of traditional frequentist statistics.

### 2.4 Model specification and prior distributions

Our analysis employed the Bayesian A/B testing framework (54): the baseline logit-transformed success rate (β) and the treatment effect as a log odds ratio (δ). The fundamental principle of Bayesian analysis is to combine experimental data with existing prior knowledge to estimate the posterior probability. The prior represents the initial beliefs or external knowledge about the different hypotheses before observing the current trial data. For the primary analysis, we specified a moderately informative normal prior, δ ~ Normal (0, 1), on the log-odds-ratio scale, which corresponds to a 95% prior interval for the odds ratio of approximately (0.14, 7.1) (55). This prior represents a neutral belief centered on no effect while still allowing clinically plausible effect sizes.

### 2.5 Sensitivity analysis

To evaluate the influence of prior choice on the BF_10_, we performed sensitivity analyses using three alternative priors: (i) a clinically concentrated normal prior, δ ~ Normal(0, 0.5), (95% prior OR interval: 0.37–2.72); (ii) a diffuse normal prior, δ ~ Normal (0, 2), (95% prior OR interval: 0.02–49.8); and (iii) the default Cauchy prior, δ ~ Cauchy(0, 0.707), as used in JASP and the BayesFactor package (95% prior OR interval: 0.03–31.6). All normal priors were implemented with the abtest package; the Cauchy prior was implemented with the BayesFactor package.

Bayes factors under normal priors were computed analytically using the abtest R package (version 1.0), which provides closed-form expressions under the Bayesian A/B testing framework. For the Cauchy prior, the BayesFactor R package (version 0.9.124.7) was used, which calculates exact analytic Bayes factors for 2 × 2 contingency tables under the independent multinomial sampling model (fixed row margins). All Bayes factors were obtained via analytic solutions.

### 2.6 Analysis of study characteristics and negative results

We first described the design features and publication trends of the included RCTs. To explore factors potentially associated with a negative trial outcome (P ≧ 0.05), we performed univariate analyses. Fisher’s exact tests were conducted for categorical variables, reporting ORs with 95% CIs. Logistic regression was performed for the continuous variable of sample size, reporting the OR per 100-participant increase. Statistical significance was assessed using two-sided tests, with a P-value < 0.05 considered statistically significant. To quantify the strength of evidence under the observed data, we also calculated the BF_10_ for each analysis. Since our aim was to reanalyze trials individually and not to derive a pooled estimate, we did not assess heterogeneity across studies.

## 3 Results

### 3.1 Bayesian reanalysis outcomes

**Figure 2:**
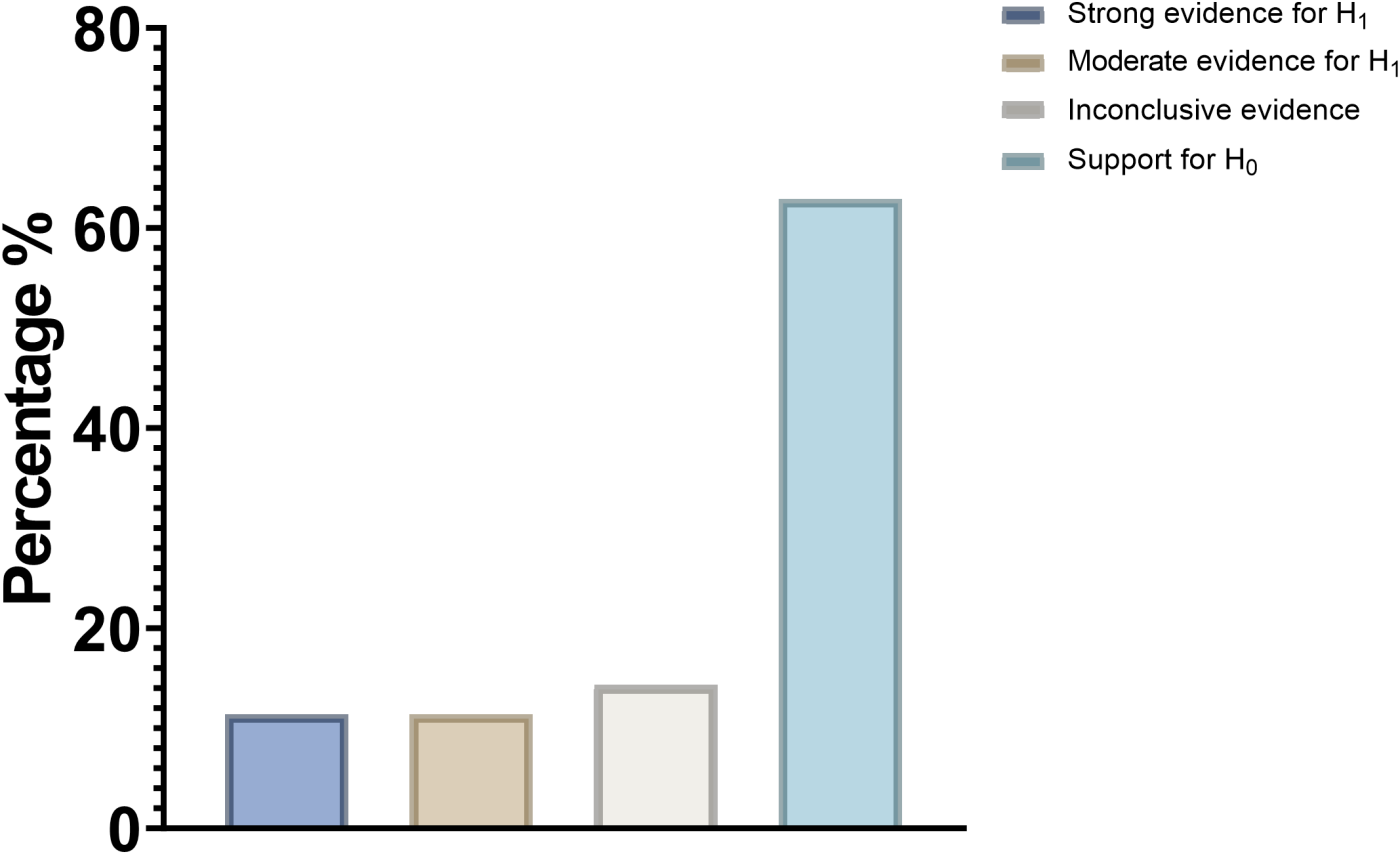
Distribution of BF_10_ across the included studies. This bar chart shows the percentage of the 35 included ART RCTs that fall into four predefined evidence-strength categories from Bayesian reanalysis. The y-axis indicates the percentage of total trials, and the x-axis represents the evidence categories. Four distinct colors correspond to the four groups, defined by Bayes Factor (BF_10_) thresholds: strong evidence for H_1_ (BF_10_ > 10), moderate evidence for H_1_ (3 < BF_10_ < 10), inconclusive evidence (1/3 < BF_10_ < 3), and support for H_0_ (BF_10_ < 1/3).

Among the 35 reanalyzed RCTs, the distribution of BF_10_ and the corresponding evidence strength classification are detailed in **Table 1**. Decisive evidence in favor of H_1_ (BF_10_ > 10) was found in four studies (11.4%); an additional four studies (11.4%) provided moderate evidence (3 < BF_10_ < 10), and five studies (14.3%) provided inconclusive evidence (1/3 < BF_10_ <3). The majority of studies (22/35, 62.9%) yielded BF_10_ < 1/3, indicating evidence in support of H_0_. The distribution of BF_10_ across all studies is shown in **Figure 2**.

**Table 1:** BF_10_ of the included studies.

Study ID	BF_10_	Evidence strength
1	0.233531	Support for H_0_
2	1.082028	Inconclusive
3	0.186094	Support for H_0_
4	0.134057	Support for H_0_
5	0.130616	Support for H_0_
6	0.255117	Support for H_0_
7	974.453462	Strong evidence for H_1_
8	0.203119	Support for H_0_
9	280.347598	Strong evidence for H_1_
10	3.424236	Moderate evidence for H_1_
11	0.201828	Support for H_0_
12	0.163986	Support for H_0_
13	0.189367	Support for H_0_
14	0.157880	Support for H_0_
15	0.229015	Support for H_0_
16	0.098300	Support for H_0_
17	0.181573	Support for H_0_
18	0.391702	Inconclusive
19	0.159759	Support for H_0_
20	0.206718	Support for H_0_
21	3.895926	Moderate evidence for H_1_
22	0.117732	Support for H_0_
23	0.220416	Support for H_0_
24	0.310898	Support for H_0_
25	1.388349	Inconclusive
26	0.988526	Inconclusive
27	0.122281	Support for H_0_
28	0.134585	Support for H_0_
29	0.169059	Support for H_0_
30	0.107124	Support for H_0_
31	122.212146	Strong evidence for H_1_
32	1.426512	Inconclusive
33	5.921177	Moderate evidence for H_1_
34	196.564251	Strong evidence for H_1_
35	6.191831	Moderate evidence for H_1_

### 3.2 Comparison between P-values and Bayes factors

A comparison of null hypothesis significance testing (NHST, based on P-values) and Bayesian analysis (based on BF_10_) is summarized in **Table 2**. The outcomes across the 35 studies were categorized into four distinct patterns: (1) concordance in supporting H_1_ (n = 8, 22.9%; significant P-values with BF_10_ > 3); (2) concordance in supporting H_0_ (n = 22, 62.9%; non-significant P-values with BF_10_ < 1/3); (3) inconclusive cases where non-significant NHST results were paired with 1/3 < BF_10_ < 3 (n = 4, 11.4%); and (4) discordant cases where statistically significant NHST results were accompanied by 1/3 < BF_10_ < 3 (n = 1, 2.9%). Overall, the two methods yielded concordant conclusions (either for H_0_ or H_1_) in 85.7% (30/35) of the studies.

**Table 2:** Comparison between NHST and Bayesian analyses.

Study ID	P-value	NHST conclusion	BF_10_	Bayes conclusion	Comparison	Classification
1	0.16	Non-significant	0.233531	Support for H_0_	Agreement	2
2	0.0007	Significant	1.082028	Inconclusive	Conflict	4
3	> 0.05	Non-significant	0.186094	Support for H_0_	Agreement	2
4	> 0.05	Non-significant	0.134057	Support for H_0_	Agreement	2
5	0.85	Non-significant	0.130616	Support for H_0_	Agreement	2
6	0.27	Non-significant	0.255117	Support for H_0_	Agreement	2
7	< 0.0001	Significant	974.453462	Strong evidence for H_1_	Agreement	1
8	0.18	Non-significant	0.203119	Support for H_0_	Agreement	2
9	0.0003	Significant	280.347598	Strong evidence for H_1_	Agreement	1
10	0.0124	Significant	3.424236	Moderate evidence for H_1_	Agreement	1
11	0.41	Non-significant	0.201828	Support for H_0_	Agreement	2
12	0.96	Non-significant	0.163986	Support for H_0_	Agreement	2
13	0.678	Non-significant	0.189367	Support for H_0_	Agreement	2
14	0.78	Non-significant	0.157880	Support for H_0_	Agreement	2
15	0.38	Non-significant	0.229015	Support for H_0_	Agreement	2
16	> 0.05	Non-significant	0.098300	Support for H_0_	Agreement	2
17	0.83	Non-significant	0.181573	Support for H_0_	Agreement	2
18	0.94	Non-significant	0.391702	Inconclusive	Conflict	3
19	0.39	Non-significant	0.159759	Support for H_0_	Agreement	2
20	0.64	Non-significant	0.206718	Support for H_0_	Agreement	2
21	0.009	Significant	3.895926	Moderate evidence for H_1_	Agreement	1
22	> 0.05	Non-significant	0.117732	Support for H_0_	Agreement	2
23	0.76	Non-significant	0.220416	Support for H_0_	Agreement	2
24*	Non-inferiority	Non-inferiority met	0.310898	Support for H_0_	Agreement	2
25	0.05	Non-significant	1.388349	Inconclusive	Conflict	3
26*	Non-inferiority	Non-inferiority met	0.988526	Inconclusive	Agreement	3
27	0.99	Non-significant	0.122281	Support for H_0_	Agreement	2
28	0.74	Non-significant	0.134585	Support for H_0_	Agreement	2
29	0.65	Non-significant	0.169059	Support for H_0_	Agreement	2
30	0.5	Non-significant	0.107124	Support for H_0_	Agreement	2
31	< 0.001	Significant	122.212146	Strong evidence for H_1_	Agreement	1
32	0.06	Non-significant	1.426512	Inconclusive	Conflict	3
33	0.004	Significant	5.921177	Moderate evidence for H_1_	Agreement	1
34	< 0.001	Significant	196.564251	Strong evidence for H_1_	Agreement	1
35	0.007	Significant	6.191831	Moderate evidence for H_1_	Agreement	1

Classification: 1. Concordant support for H_1_; 2. Concordant support for H_0_; 3. Non-significant P-value with inconclusive BF_10_ (1/3 < BF_10_ < 3); 4. Significant P-value with inconclusive BF_10_ (1/3 < BF_10_ < 3). *For non-inferiority trials (ID = 24 and ID = 26), the Bayes factors were computed under a superiority framework (testing for any difference). A BF_10_ < 3 indicates an absence of evidence for superiority, which is consistent with the original non-inferiority conclusions.

### 3.3 Sensitivity analysis

To assess the robustness of the results with respect to the prior specification, we compared the primary Normal(0,1) prior against three alternatives: a clinically calibrated Normal(0,0.5) prior, a diffuse Normal(0,2) prior, and the community-standard Cauchy(0,0.707) prior. The three normal priors (σ = 1, 0.5, and 2) yielded identical evidential classifications (support for H_0_, inconclusive evidence, or support for H_1_) in all 35 trials. However, when using the Cauchy(0,0.707) prior, three trials (8.6%) showed different classifications compared to the normal priors (**Table 3**). In these three trials, the Cauchy prior shifted the classification from “support for H_1_” to “inconclusive” (two trials) or from “inconclusive” to “support for H_0_” (one trial), with the Bayes factors lying close to the predefined thresholds (BF_10_ ≈ 2.3 vs. 3, and BF_10_ ≈ 0.10 vs. 0.33). Crucially, no trial shifted from support for H_0_ to support for H_1_ (or vice versa) under any prior. The overall conclusions of the study remain unchanged, and the observed sensitivity was confined to borderline cases where the data provided weak evidence.

**Table 3:** Sensitivity analysis: BF_10_ under alternative prior distributions.

Study ID	BF_10_ [Primary Prior: δ ~ N(0, 1))	BF_10_ [Concentrated Prior: δ ~ N(0, 0.5)]	BF_10_ [Diffuse Prior: δ ~ N(0, 2)]	BF_10_ [Prior:δ ~ Cauchy(0, 0.707)]
1	0.2335	0.2329	0.2337	0.1347
2	1.0820	0.8547	1.2130	0.4665
3	0.1861	0.1857	0.1862	0.1078
4	0.1341	0.1339	0.1341	0.0752
5	0.1306	0.1306	0.1306	0.0824
6	0.2551	0.2542	0.2553	0.1434
7	974.4535	973.1181	974.7920	649.8897
8	0.2031	0.2023	0.2033	0.0997
9	280.3476	177.6829	324.1287	275.6930
10	3.4242	3.4246	3.4242	2.2779
11	0.2018	0.2018	0.2018	0.1271
12	0.1640	0.1634	0.1641	0.0858
13	0.1894	0.1894	0.1894	0.1202
14	0.1579	0.1577	0.1579	0.0944
15	0.2290	0.2289	0.2291	0.1397
16	0.0983	0.0982	0.0983	0.0560
17	0.1816	0.1795	0.1821	0.0686
18	0.3917	0.3646	0.4012	0.1035
19	0.1598	0.1586	0.1601	0.0699
20	0.2067	0.2067	0.2067	0.1312
21	3.8959	3.8592	3.9055	2.3867
22	0.1177	0.1177	0.1177	0.0719
23	0.2204	0.2186	0.2209	0.1155
24	0.3109	0.3109	0.3109	0.2002
25	1.3883	1.3787	1.3910	0.8980
26	0.9885	0.9610	0.9958	0.4218
27	0.1223	0.1220	0.1224	0.0595
28	0.1346	0.1345	0.1346	0.0798
29	0.1691	0.1688	0.1691	0.0980
30	0.1071	0.1071	0.1071	0.0674
31	122.2121	120.0894	122.7622	75.8329
32	1.4265	1.3974	1.4343	0.8220
33	5.9212	5.9226	5.9208	3.8191
34	196.5643	105.5941	238.9269	205.4614
35	6.1918	5.8774	6.2777	3.0853

### 3.4 Publication trends and study design features

The characteristics of the included studies are detailed in **Table 4**. Overall, 71.4% (25/35) of the studies reported statistically non-significant (“negative”) primary outcomes. This proportion increased from 60% (2010–2015) to 72.22% (2016–2020) and remained at a high level at 75% (2021–2025), showing an upward trend. However, the proportion of large-scale trials (sample size ≥ 700) rose markedly from 20.0% to 83.3% across the same periods. The majority of trials employed superiority designs (94.3%), two-arm designs (80.0%), active-treatment comparators (68.6%), and evaluated therapeutic interventions (74.3%).

**Table 4:** Publication trends and design characteristics of the included studies.

		Year of publication			all
Characteristic	Subgroup	2010–2015	2016–2020	2021–2025	
		N(%)	N(%)	N(%)	
		N = 5	N = 18	N = 12	N = 35
Results	Positive	2 (40)	5 (27.78)	3 (25)	10 (28.57)
	Negative	3 (60)	13 (72.22)	9 (75)	25 (71.43)
Journal	The British Medical Journal	2 (40)	1 (5.56)	3 (25)	6 (17.14)
	The Journal of the American Medical Association	0 (0)	7 (38.89)	2 (16.67)	9 (25.71)
	The Lancet	1 (20)	6 (33.33)	6 (50)	13 (37.14)
	The New England Journal of Medicine	2 (40)	4 (22.22)	1 (8.33)	7 (20)
Study design	Superiority trial	4 (80)	18 (100)	12 (100)	33 (94.29)
	Equivalence or non‐inferiority	1 (20)	0 (0)	1 (8.33)	2 (5.71)
Number of arms	Two	3 (60)	16 (88.89)	9 (75)	28 (80)
	More than two	2 (40)	2 (11.11)	3 (25)	7 (20)
Control group	Placebo/No treatment	1 (20)	7 (38.89)	3 (25)	11 (31.43)
	Treatment	4 (80)	11 (61.11)	9 (75)	24 (68.57)
Type of intervention	Drug	2 (40)	4 (22.22)	1 (8.33)	7 (20)
	Medical device	0 (0)	1 (5.56)	1 (8.33)	2 (5.71)
	Therapeutic strategy	3 (60)	13 (72.22)	10 (83.33)	26 (74.29)
Number of patients randomized	< 700	4 (80)	5 (27.78)	2 (16.67)	11 (31.43)
	≧ 700	1 (20)	13 (72.22)	10 (83.33)	24 (68.57)

This table summarizes the design features and outcome directions of the included RCTs, stratified by three publication periods: 2010–2015, 2016–2020, and 2021–2025. Data are presented as the number (percentage) of trials within each category. “Positive” results are defined as a statistically significant P-value (P < 0.05) for the primary outcome; “Negative” results are defined as a non-significant P-value (P ≥ 0.05) for the primary outcome.

Univariate associations of factors associated with negative results are detailed in **Table 5**. The overall Fisher’s exact test for journal differences showed no significant association (P = 0.172, BF_10_ = 0.810). Although not statistically significant (P ≧ 0.05), several consistent patterns were observed. The proportion of negative results was numerically lower in NEJM (50.0%, 5/10) than in the other three journals (69.2%–100.0%). In separate comparisons, the odds ratio for JAMA compared with NEJM was 0.077 (95% CI: 0.003–1.724, BF_10_ ≈ 2.59), with the Bayes factor indicating weak but consistent evidence in favor of a positive association. A larger sample size (≧ 500) was associated with a higher likelihood of a negative outcome (74.1% vs. 62.5%; OR = 1.739, 95% CI 0.358–8.443). Live birth or related outcomes constituted the primary endpoint in the majority of trials (74.3%, 26/35), with both methods suggesting evidence in favor of no difference (P = 0.6936, BF_10_ ≈ 0.35). Other design features also showed no significant associations.

**Table 5:** Factors associated with negative trial results.

Characteristic	Subgroup	Negative trials/Total (%)	OR (95% CI)	P-Value	BF_10_
Overall	NA	25/35 (71.4%)	NA	NA	NA
Journal	Overall	NA	NA	0.172	0.810
	NEJM*	5/10 (50.0%)	1	NA	NA
	The Lancet	9/13 (69.2%)	0.474 (0.093–2.416)	0.4173	0.58
	JAMA	6/6 (100%)	0.077 (0.003–1.724)	0.0934	2.59
	BMJ	5/6 (83.3%)	0.273 (0.032–2.360)	0.3069	0.79
Sample Size	Per 100-patient increase	NA	0.931 (0.816–1.061)	0.2825	0.86
	≥ 500	3/5 (62.5%)	1.739 (0.358–8.443)	0.6614	0.37
	< 500	7/20 (74.1%)			
	< 400	2/5 (40.0%)	NA	0.2011	1.2
	400–800	10/12 (83.3%)			
	≧ 800	13/18 (72.2%)			
Primary Outcome	Live birth (or related)	19/26 (73.1%)	0.714 (0.152–3.360)	0.6936	0.35
	Others	6/9 (66.7%)			
Trial Design	Multicenter	23/33 (69.7%)	2.234 (0.098–50.713)	1	0.24
	Single-center	2/2 (100.0%)			
	Double-blind	11/13 (84.6%)	0.371 (0.074–1.853)	0.2591	0.93
	Open-label	14/22 (63.6%)			
	Parallel	20/29 (69.0%)	1.699 (0.239–12.093)	0.6493	0.37
	Non-parallel	5/6 (83.3%)			

ORs with 95% CIs were calculated using 2 × 2 contingency tables (categorical variables) or logistic regression (continuous variables). P-values were derived from Fisher’s exact tests (categorical variables) or Wald tests (continuous variables). BF_10_ values were computed as described in the Methods section. *refers to the reference category used in the calculation.

## 4 Discussion

### 4.1 Implications of the study

This study demonstrates that Bayesian statistical methods provide a quantitative complement to conventional frequentist statistics for interpreting negative results in ART RCTs. While the majority of researchers apply P-values to assess result uncertainty, Bayes factors offer a standardized metric for quantifying the strength of evidence for both the null and alternative hypotheses (55).

A critical advantage of the Bayesian approach is its ability to formally distinguish between “absence of evidence” and “evidence of absence”. In NHST, a non-significant P-value is frequently mistaken for evidence supporting H_0_ (53). However, such a result may simply reflect an insufficient sample size or high variability rather than genuine support for H_0_. In contrast, Bayesian analysis directly addresses this ambiguity: for instance, a BF_10_ < 1/3 provides evidence of absence, indicating that the data strongly favor H_0_, while a BF_10_ ≈ 1 represents an absence of evidence, meaning the data are insufficient to meaningfully distinguish between the hypotheses. This distinction helps avoid equating non-significance with a conclusion of no effect, thereby guiding more informed clinical and research decisions.

The application of Bayes factors provides a critical refinement to the interpretation of non-significant NHST results by distinguishing two distinct cases: (i) evidence of absence, as seen in the RCT evaluating hysteroscopy in IVF, where a non-significant result was accompanied by a BF_10_ of 0.16 (17), suggesting that larger confirmatory trials may not be necessary; and (ii) inconclusive data, as seen in the lifestyle trial by Mutsaerts et al., where a similarly non-significant P-value corresponded to a BF_10_ of 1.42, indicating weak evidence and data insensitivity due to insufficient sample size (18). The latter suggests that further research is required to clarify the intervention effect (56).

Furthermore, a minor divergence between methods was observed in one study, where significant NHST results were inconsistent with inconclusive Bayesian conclusions (BF_10_ ≈ 1). This discordance can be explained by fundamental methodology: false-positive results are more likely to occur when the sample size is large or the effect size is small. For this study, BF_10_ ≈ 1 indicates that the data had weak discriminatory power, demonstrating that significant NHST results do not necessarily equate to substantive evidential support for H_1_ (57).

Our findings demonstrate that while Bayesian methods provide a superior framework for evidential interpretation, their conclusions are broadly consistent with NHST results, which aligns with the results of similar reanalyses conducted in other medical fields (14–16). This concordance suggests that when NHST yields clear results, Bayesian methods often lead to consistent conclusions, thereby further enhancing credibility. Therefore, Bayesian analysis should be considered a powerful complement to the established frequentist framework, particularly for interpreting ambiguous results, rather than a replacement (53).

It is crucial to emphasize that Bayes factors reflect only the strength of statistical evidence, not clinical significance. It is entirely possible to obtain a BF_10_ > 3 (moderate evidence for H_1_) while observing an effect size smaller than the minimal clinically important difference (MCID) (14). In such cases, the results may be statistically discernible but clinically irrelevant. Therefore, clinical decisions should integrate effect-size estimates, evidence strength, and clinical context.

Exploratory univariate analysis revealed several trends associated with negative trial outcomes. The observed variation in the proportion of negative results across journals may reflect the general situation of research during this period rather than a definitive editorial policy. Statistical results indicated an association between sample size and the probability of a negative result, which showed a similar trend to that observed in other fields of research (58).

This trend carries important methodological implications. Larger RCTs possess greater statistical power to detect smaller, clinically realistic effect sizes and are therefore more likely to yield a definitive negative conclusion when an intervention is truly ineffective. In contrast, the higher proportion of positive results among smaller RCTs may indicate that underpowered studies are more susceptible to false-positive findings due to random error. This finding underscores the importance of adequate power and sample size calculation in the design phase of RCTs. The widespread adoption of live birth as the primary outcome reflects methodological maturity in the field. However, the high overall rate of negative results also suggests a key reality: under current technological paradigms, the absolute treatment effects of many interventions aimed at improving live birth rates are likely limited.

In summary, our secondary analysis reveals that the majority of RCTs in this field have sufficient statistical power to provide definitive evidence supporting H_0_ rather than merely inconclusive data. This finding suggests that future research should prioritize the development and evaluation of novel intervention targets rather than repeating trials of interventions that have already been shown to have no meaningful benefit. By continuously assessing evidence strength, Bayesian analysis advances the field from merely assessing “whether an intervention is effective” to evaluating “how strong the evidence is,” thereby providing a robust methodological foundation for the design and interpretation of future clinical trials.

### 4.2 Advantages and limitations

The primary strength of this study lies in its systematic application of Bayesian methods to reinterpret “negative” ART trials, offering a quantitative evidence framework that complements traditional P-value-based inference. However, there are still some limitations and challenges that should be interpreted with caution.

First, our search was restricted to four high-impact general medical journals to select for trials of high methodological and clinical relevance. While this ensures a robust evidence base for our reanalysis, it introduces selection bias and limits the generalizability of our findings to the broader ART literature. Caution should be exercised when extending interpretations to studies with different designs or with a higher risk of bias.

Another key consideration pertains to the Bayesian methodology itself. The ability of the Bayesian approach to combine prior information with current data is both a feature and an inherent limitation, as it introduces high dependency on the prior distribution. This dependency directly influences the magnitude, direction, and robustness of statistical conclusions, a concern substantiated by empirical research (53, 59, 60). Transparent reporting and justification of priors are essential for minimizing subjective bias and ensuring reproducibility. Our sensitivity analysis across four priors showed that the three normal priors were fully consistent, and the only discrepancies occurred with the Cauchy prior in three borderline cases, without any directional reversal, further confirming the robustness of our conclusions.

Second, BF_10_ ≈ 1 indicates data insensitivity, reflecting the limitations of the original trial design, such as a small sample size and high variability. These cases underscore that neither NHST nor Bayesian methods can overcome fundamental data limitations (61). The observed discrepancies in these cases should be interpreted primarily as a reflection of methodological differences rather than definitive clinical conclusions. Therefore, regardless of the analytical method, these results require cautious interpretation to avoid overstatement and misinterpretation.

Our analysis computed BF_10_ under a superiority framework, testing the null hypothesis of no difference against the alternative of any difference. For the two non-inferiority trials included (ID = 24 and 26), the original frequentist hypotheses are different: the null is that the experimental treatment is inferior by at least a prespecified margin, and the alternative is non-inferiority. A Bayes factor designed for non-inferiority would require specifying a prior distribution that incorporates the non-inferiority margin, which is beyond the scope of this study. However, the BF_10_ values we obtained indicate that the data do not provide strong evidence of superiority, which is fully compatible with the original non-inferiority conclusions. Therefore, while our Bayes factors are not direct tests of non-inferiority, they do not contradict the original findings.

Finally, it is crucial to distinguish statistical evidence from clinical relevance. Clinical decision-making must integrate multidimensional considerations, including MCID, safety assessment, cost-effectiveness, and patient preferences, which extend beyond statistical outputs. Therefore, while this study provides methodological insights, its practical clinical value still needs to be further demonstrated by follow-up studies (62).

In conclusion, both advantages and challenges of Bayesian methods exist. While NHST retains advantages in standardization, Bayesian methods should be viewed as complementary tools. Adopting a dual-track approach combining the strengths of both paradigms can enhance the explanatory power and clinical applicability of statistical conclusions.

### 4.3 Future research directions

Future research can be further expanded and optimized in the following aspects.

First, to improve generalizability and evidential strength, future RCTs should prioritize multicenter designs with adequate sample sizes, which will also enhance the stability of both NHST and Bayesian methods (63).

Second, reliance on prior distributions can be transformed into an advantage by enhancing the explanatory power of analysis with scientifically informed priors (64). We recommend developing an evidence-based “prior knowledge repository” for ART, built from real-world data and expert consensus, to strengthen the reproducibility and predictive utility of Bayesian models.

Third, the increasing availability of large-scale clinical databases offers an opportunity to develop more powerful predictive models. Bayesian methods, particularly Bayesian networks, are especially suitable for modeling the complex ART data. Their integration with machine learning could support personalized prognosis and treatment selection (65). As preliminarily demonstrated in our previous work, we have successfully developed a predictive Bayesian network model for fertilization disorders in infertility, integrating multivariate clinical factors to offer clinical recommendations for ART strategies (66).

In summary, future research should continue to promote the application of Bayesian statistics in three interconnected dimensions: methodological optimization, prior construction, and model integration. By fostering collaboration with multidisciplinary technology, the Bayesian method can provide high-quality evidence and intelligent decision-making tools for improving clinical outcomes.

## 5 Conclusion

This Bayesian reanalysis of high-quality ART RCTs demonstrates that a substantial proportion of statistically non-significant results actually provide evidence supporting H_0_, while also identifying truly inconclusive trials. By quantifying evidence strength, Bayesian methods effectively complement NHST, offering a valuable supplementary tool for interpreting trial outcomes and guiding future research design in reproductive medicine.

## Data Availability

The data supporting the findings of this study are derived from 35 publicly available randomized controlled trials, all of which are accessible via PubMed and fully cited in references of this manuscript. No new primary clinical data were generated for this secondary methodological reanalysis. The custom R code used for all Bayesian re-analyses is available from the corresponding authors upon reasonable request.
