# Supplementary material for: A Bayesian reevaluation of randomized controlled trials in assisted reproductive technology: quantifying evidence strength for null and alternative hypotheses
Source: Front Endocrinol (Lausanne). 2026 Jun 12;17:1860725. doi: 10.3389/fendo.2026.1860725 (PMC13303236; doi:10.3389/fendo.2026.1860725)
Supplement: Supplementary file 1 [file DataSheet1.docx]

Supplementary Material

# Supplementary Figures


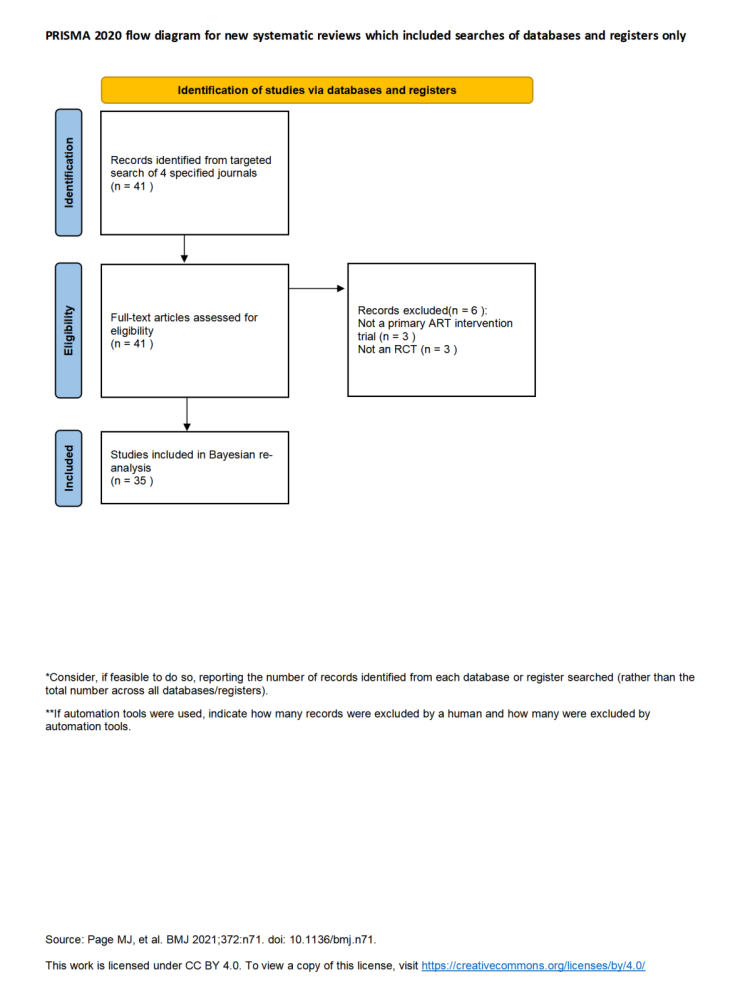


Supplementary Figure 1. Study Selection Flow Diagram


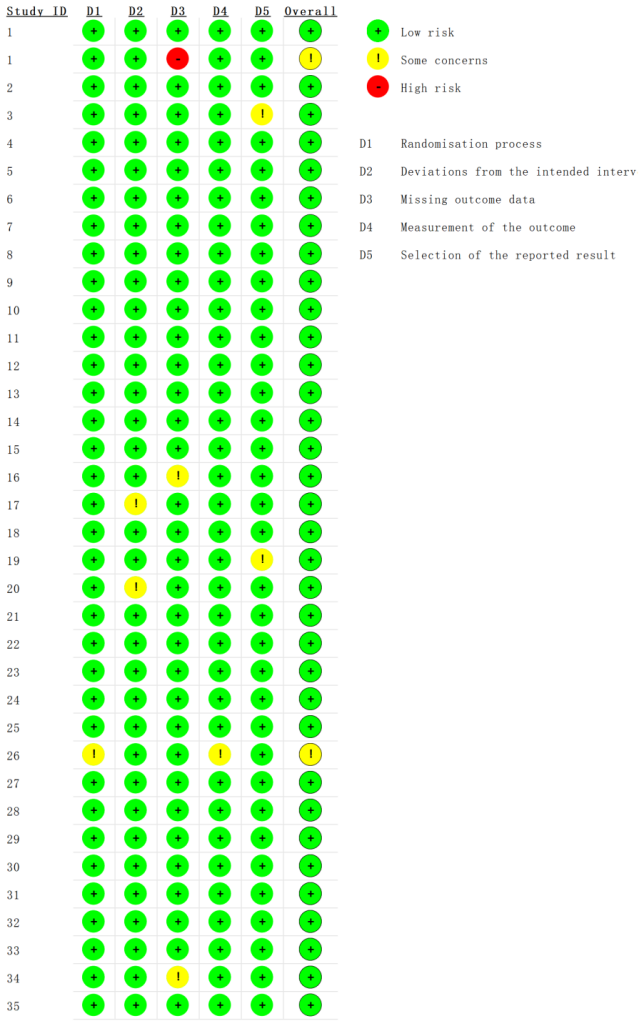


Supplementary Figure 2. Risk Of Bias Assessment Summary For The Included Randomized Trials

# Supplementary Tables

| Study ID | Title | Study Design | Journal | Time | Region | First Author | Primary Outcome | Intervention 1 | Intervention 2 | Intervention 3 | Analytical Method |
| --- | --- | --- | --- | --- | --- | --- | --- | --- | --- | --- | --- |
| 1 | Intracytoplasmic sperm injection versus conventional in-vitro fertilisation for couples with infertility with non-severe male factor: a multicentre, open-label, randomised controlled trial | multicentre,open-label,randomised controlled trial | Lancet | 2024 | China | Wang Yuanyuan | live birth after first embryo transfer | ICSI | IVF | NA | ITT |
| 2 | Bariatric surgery for spontaneous ovulation in women living with polycystic ovary syndrome: the BAMBINI multicentre, open-label, randomised controlled trial | multicentre,open-label,randomised controlled trial | Lancet | 2024 | UK | Suhaniya N S Samarasinghe | the number of biochemically confirmed ovulatory events over 52 weeks | vertical sleeve gastrectomy | behavioural interventions and medical therapy | NA | ITT |
| 3 | Livebirth rate after one frozen embryo transfer in ovulatory women starting with natural, modified natural, or artificial endometrial preparation in Viet Nam: an open-label randomised controlled trial | three-parallel-group,single-center,open-label,randomised controlled trial | Lancet | 2024 | Vietnam | Vu N A Ho | livebirth after one FET | natural cycle | artificial cycle | modified cycle | ITT |
| 4 | Clinical effectiveness and safety of time-lapse imaging systems for embryo incubation and selection in in-vitro fertilisation treatment (TILT): a multicentre, three-parallel-group, double-blind, randomised controlled trial | three-parallel-group,multicentre,double-blind, randomised controlled trial | Lancet | 2024 | UK | Priya Bhide | live birth | time-lapse imaging | standard care | undisturbed culture | ITT |
| 5 | Clinical outcomes of uninterrupted embryo culture with or without time-lapse-based embryo selection versus interrupted standard culture (SelecTIMO): a three-armed, multicentre, double-blind, randomised controlled trial | three-parallel-group,multicentre,double-blind, randomised controlled trial | Lancet | 2023 | Netherlands | D C Kieslinger | cumulative ongoing pregnancy rate within 12 months | time-lapse early embryo viability assessment | control | time-lapse routine | ITT |
| 6 | Intracytoplasmic sperm injection versus conventional in-vitro fertilisation in couples with infertility in whom the male partner has normal total sperm count and motility: an open-label, randomised controlled trial | multicentre,open-label,randomised controlled trial | Lancet | 2021 | Vietnam | Vinh Q Dang | livebirth after the first embryo transfer from the initiated cycle | ICSI | IVF | NA | ITT |
| 7 | Frozen versus fresh single blastocyst transfer in ovulatory women: a multicentre, randomised controlled trial | multicentre,open-label,randomised controlled trial | Lancet | 2019 | China | Daimin Wei | singleton livebirth rate | fresh single blastocyst transfer | frozen single blastocyst transfer | NA | ITT |
| 8 | Physiological, hyaluronan-selected intracytoplasmic sperm injection for infertility treatment (HABSelect): a parallel, two-group, randomised trial | double-blind,multicenter,randomized controlled trial | Lancet | 2019 | UK | David Miller | full-term (≥37 weeks' gestational age) livebirth | PICSI | ICSI | NA | ITT |
| 9 | Intrauterine insemination with ovarian stimulation versus expectant management for unexplained infertility (TUI): a pragmatic, open-label, randomised, controlled, two-centre trial | multicenter,open-label,randomized controlled trial | Lancet | 2018 | New Zealand | Prof Cynthia M Farquhar | cumulative livebirth rate | three cycles of IUI | three cycles of expectant management | NA | ITT |
| 10 | Gonadotrophins versus clomifene citrate with or without intrauterine insemination in women with normogonadotropic anovulation and clomifene failure (M-OVIN): a randomised, two-by-two factorial trial | multicenter,open-label,two-by-two factorial,randomised controlled trial | Lancet | 2018 | Netherlands | Nienke S Weiss | conception leading to livebirth within 8 months after randomisation | gonadotrophins | clomifene citrate | NA | ITT |
| 11 | Hysteroscopy before in-vitro fertilisation (inSIGHT): a multicentre, randomised controlled trial | multicenter,open-label,randomized controlled trial | Lancet | 2016 | Netherlands | Janine G Smit | ongoing pregnancy within 18 months of randomisation and resulting in livebirth | hysteroscopy | mmediate IVF | NA | ITT |
| 12 | Hysteroscopy in recurrent in-vitro fertilisation failure (TROPHY): a multicentre, randomised controlled trial | double-blind,multicenter,randomized controlled trial | Lancet | 2016 | UK | Dr Tarek El-Toukhy MRCOG | livebirth rate | outpatient hysteroscopy | no hysteroscopy | NA | ITT |
| 13 | Salpingotomy versus salpingectomy in women with tubal pregnancy (ESEP study): an open-label, multicentre, randomised controlled trial | multicenter,open-label,randomized controlled trial | Lancet | 2014 | Netherlands | Femke Mol | ongoing pregnancy by natural conception | salpingotomy | salpingectomy | NA | ITT |
| 14 | Prednisone vs Placebo and Live Birth in Patients With Recurrent Implantation Failure Undergoing In Vitro Fertilization: A Randomized Clinical Trial | double-blind,multicenter,randomized controlled trial | JAMA | 2023 | China | Yun Sun | live birth | 10 mg of prednisone | placebo | NA | ITT |
| 15 | Effect of Timing by Endometrial Receptivity Testing vs Standard Timing of Frozen Embryo Transfer on Live Birth in Patients Undergoing In Vitro Fertilization: A Randomized Clinical Trial | double-blind,multicenter,randomized controlled trial | JAMA | 2022 | USA | Nicole Doyle | live birth | endometrial receptivity testing | standardized timing | NA | ITT |
| 16 | Effect of Folic Acid and Zinc Supplementation in Men on Semen Quality and Live Birth Among Couples Undergoing Infertility Treatment: A Randomized Clinical Trial | double-blind,multicenter,randomized controlled trial | JAMA | 2020 | USA | Enrique F. Schisterman | live birth | 5 mg of folic acid and 30 mg of elemental zinc | placebo | NA | ITT |
| 17 | Effect of Acupuncture vs Sham Acupuncture on Live Births Among Women Undergoing In Vitro Fertilization: A Randomized Clinical Trial | double-blind,multicenter,randomized controlled trial | JAMA | 2018 | Australia | Caroline A. Smith | live birth | acupuncture | sham acupuncture | NA | ITT |
| 18 | Effect of Levothyroxine on Miscarriage Among Women With Normal Thyroid Function and Thyroid Autoimmunity Undergoing In Vitro Fertilization and Embryo Transfer: A Randomized Clinical Trial | single-center,open-label,randomised controlled trial | JAMA | 2017 | China | Haining Wang | miscarriage rate | 25-μg/d or 50-μg/d dose of levothyroxine | control | NA | ITT |
| 19 | Effect of Acupuncture and Clomiphene in Chinese Women With Polycystic Ovary Syndrome  A Randomized Clinical Trial | double-blind,multicenter,two-by-two factorial, randomised controlled trial | JAMA | 2017 | China | Xiao-Ke Wu | live birth | Active Acupuncture | control acupuncture | NA | ITT |
| 20 | Preimplantation genetic testing for aneuploidy versus no genetic testing in couples undergoing intracytoplasmic sperm injection for severe male infertility: multicentre, open label, randomised controlled trial | Multicentre,open label,randomised controlled trial | BMJ | 2025 | China | Xianhua Lin | live birth after the first embryo transfer | ICSI with PGT-A | ICSI | NA | ITT |
| 21 | Frozen versus fresh embryo transfer in women with low prognosis for in vitro fertilisation treatment: pragmatic, multicentre, randomised controlled trial | multicenter,open-label,randomized controlled trial. | BMJ | 2025 | China | Daimin Wei | live birth | frozen embryo transfer | fresh embryo transfer | NA | ITT |
| 22 | Cumulative live birth rate of a blastocyst versus cleavage stage embryo transfer policy during in vitro fertilisation in women with a good prognosis: multicentre randomised controlled trial | multicenter,open-label,randomized controlled trial. | BMJ | 2024 | Netherlands | Simone Cornelisse | the cumulative live birth rate per oocyte retrieval | blastocyst stage embryo transfer | cleavage stage embryo transfer | NA | ITT |
| 23 | Freeze-all versus fresh blastocyst transfer strategy during in vitro fertilisation in women with regular menstrual cycles: multicentre randomised controlled trial | double-blind,multicenter,randomized controlled trial | BMJ | 2020 | Denmark | Sacha Stormlund | the ongoing pregnancy rate | freeze-all | fresh transfer | NA | ITT |
| 24 | Prevention of multiple pregnancies in couples with unexplained or mild male subfertility: randomised controlled trial of in vitro fertilisation with single embryo transfer or in vitro fertilisation in modified natural cycle compared with intrauterine insemination with controlled ovarian hyperstimulation | multicentre,open label,three-parallel-group,randomised controlled non-inferiority trial | BMJ | 2015 | Netherlands | A J Bensdorp | birth of a healthy child resulting from a singleton pregnancy conceived within 12 months after randomisation | in vitro fertilisation with single embryo transfer | intrauterine insemination with controlled ovarian hyperstimulation | in vitro fertilisation in a modified natural cycle | ITT |
| 25 | The effect of a multifaceted empowerment strategy on decision making about the number of embryos transferred in in vitro fertilisation: randomised controlled trial | multicenter,open-label, randomized controlled trial | BMJ | 2010 | Netherlands | Arno van Peperstraten | Use of single embryo transfer in the first cycle | multifaceted strategy | control | NA | ITT |
| 26 | Live Birth with or without Preimplantation Genetic Testing for Aneuploidy | multicenter,open-label,randomized controlled non-inferiority trial | NEJM | 2021 | China | Junhao Yan | the cumulative live-birth rate after up to three embryo-transfer procedures within 1 year after randomization | PGT-A | IVF | NA | ITT |
| 27 | A Randomized Trial of Endometrial Scratching before In Vitro Fertilization | multicenter,open-label,randomized controlled trial | NEJM | 2019 | New Zealand | Sarah Lensen | live birth | endometrial scratching | control | NA | ITT |
| 28 | Levothyroxine in Women with Thyroid Peroxidase Antibodies before Conception | double-blind,multicenter,randomized controlled trial | NEJM | 2019 | UK | Rima K Dhillon-Smith | live birth after at least 34 weeks of gestation | 50 μg once daily of levothyroxine | placebo | NA | ITT |
| 29 | IVF Transfer of Fresh or Frozen Embryos in Women without Polycystic Ovaries | multicenter,open-label,randomized controlled trial | NEJM | 2018 | Vietnam | Lan N. Vuong | ongoing pregnancy after the first embryo transfer | frozen embryo | fresh embryo | NA | ITT |
| 30 | Transfer of Fresh versus Frozen Embryos in Ovulatory Women | multicenter,open-label,randomized controlled trial | NEJM | 2018 | China | Yuhua Shi | live birth after the first embryo transfer | frozen-embryo | fresh-embryo | NA | ITT |
| 31 | Oil-Based or Water-Based Contrast for Hysterosalpingography in Infertile Women | multicenter,open-label,randomized controlled trial | NEJM | 2017 | Netherlands | Kim Dreyer | ongoing pregnancy within 6 months after randomization | oil contrast | water contrast | NA | ITT |
| 32 | Randomized Trial of a Lifestyle Program in Obese Infertile Women | ,open-label,randomized controlled trial | NEJM | 2016 | Netherlands | Meike A.Q. Mutsaerts | the vaginal birth of a healthy singleton at term within 24 months after randomization | 6-month lifestyle-intervention preceding treatment | prompt treatment | NA | ITT |
| 33 | Fresh versus Frozen Embryos for Infertility in the Polycystic Ovary Syndrome | multicenter,open-label,randomized controlled trial | NEJM | 2016 | China | Zi-Jiang Chen | live birth after the first embryo transfer | frozen-embryo transfer | fresh-embryo transfer | NA | ITT |
| 34 | Letrozole, Gonadotropin, or Clomiphene for Unexplained Infertility | double-blind,multicenter,randomized controlled trial | NEJM | 2015 | USA | Michael P. Diamond | the rate of multiple gestations among women with clinical pregnancies | Gonadotropin | Clomiphene | Letrozole | ITT |
| 35 | Letrozole versus clomiphene for infertility in the polycystic ovary syndrome | double-blind,multicenter,randomized controlled trial | NEJM | 2014 | USA | Richard S. Legro | live birth during the treatment period | letrozole | clomiphene | NA | ITT |

Supplementary Table 1. Study Identification of included studies

| Study ID | Event Count 1 | Group Number 1 | Incidence Rate 1 | Event Count 2 | Group Number 2 | Incidence Rate 2 | Event Count 3 | Group Number 3 | Incidence Rate 3 | P-  value | RR | IRR | OR | 95%CI |
| --- | --- | --- | --- | --- | --- | --- | --- | --- | --- | --- | --- | --- | --- | --- |
| 1 | 390 | 1154 | 33.80% | 430 | 1175 | 36.60% | NA | NA | NA | 0.16 | 0.92 | NA | NA | 0.83-1.03 |
| 2 | 6 | 40 | 15% | 2 | 40 | 5% | NA | NA | NA | 0.0007 | NA | 2.5 | NA | 1.5-4.2 |
| 3 | 174 | 476 | 37% | 162 | 476 | 34% | 159 | 476 | 33% | >0.05 | 1.07 | NA | NA | 0.87-1.33 |
| 4 | 175 | 520 | 33.70% | 172 | 522 | 33% | 189 | 516 | 36.60% | >0.05 | NA | NA | 1.04 | 0.73 -1.47 |
| 5 | 293 | 577 | 50.80% | 284 | 575 | 49.40% | 295 | 579 | 50.90% | 0.85 | NA | NA | 1.06 | 0.84-1.33 |
| 6 | 184 | 532 | 35% | 166 | 532 | 31% | NA | NA | NA | 0.27 | 1.11 | NA | NA | 0.93 - 1.32 |
| 7 | 329 | 825 | 40% | 416 | 825 | 50% | NA | NA | NA | <0.0001 | 1.26 | NA | NA | 1.14-1.41 |
| 8 | 379 | 1381 | 27.40% | 346 | 1371 | 25.20% | NA | NA | NA | 0.18 | 1.12 | NA | NA | 0.95-1.34 |
| 9 | 31 | 101 | 31% | 9 | 100 | 9% | NA | NA | NA | 0.0003 | 3.41 | NA | NA | 1.71-6.79 |
| 10 | 167 | 327 | 52% | 138 | 334 | 41% | NA | NA | NA | 0.0124 | 1.24 | NA | NA | 1.05-1.46 |
| 11 | 209 | 373 | 57% | 200 | 377 | 54% | NA | NA | NA | 0.41 | 1.06 | NA | NA | 0.93-1.20 |
| 12 | 102 | 350 | 29% | 102 | 352 | 29% | NA | NA | NA | 0.96 | 1 | NA | NA | 0.79-1.25 |
| 13 | 108 | 215 | 60.70% | 114 | 231 | 56.20% | NA | NA | NA | 0.678 | 1.06 | NA | NA | 0.81-1.38 |
| 14 | 135 | 357 | 37.80% | 139 | 358 | 38.80% | NA | NA | NA | 0.78 | 0.97 | NA | NA | 0.81-1.17 |
| 15 | 223 | 381 | 58.50% | 239 | 386 | 61.90% | NA | NA | NA | 0.38 | 0.95 | NA | NA | 0.79-1.13 |
| 16 | 404 | 1185 | 34% | 416 | 1185 | 35% | NA | NA | NA | >0.05 | 0.98 | NA | NA | 0.88-1.09 |
| 17 | 74 | 405 | 18.30% | 72 | 404 | 17.80% | NA | NA | NA | 0.83 | 1.02 | NA | NA | 0.76-1.38 |
| 18 | 11 | 107 | 10.30% | 12 | 113 | 10.60% | NA | NA | NA | 0.94 | 0.97 | NA | NA | 0.45-2.10 |
| 19 | 100 | 458 | 21.80% | 105 | 468 | 22.40% | NA | NA | NA | 0.39 | NA | NA | NA | -5.9%-4.7% |
| 20 | 109 | 225 | 48.40% | 104 | 225 | 46.20% | NA | NA | NA | 0.64 | 1.09 | NA | NA | 0.76 - 1.58 |
| 21 | 132 | 419 | 32.00% | 168 | 419 | 40.00% | NA | NA | NA | 0.009 | 0.79 | NA | NA | 0.65- 0.94 |
| 22 | 355 | 603 | 58.90% | 350 | 599 | 58.40% | NA | NA | NA | >0.05 | 1.01 | NA | NA | 0.84-1.22 |
| 23 | 62 | 223 | 27.80% | 68 | 230 | 29.60% | NA | NA | NA | 0.76 | 0.98 | NA | NA | 0.87 - 1.10 |
| 24 | 104 | 201 | 52% | 97 | 207 | 47% | 83 | 194 | 43% | >0.05 | 1.1 | NA | NA | 0.91 - 1.34 |
| 25 | 65 | 152 | 43% | 50 | 156 | 32% | NA | NA | NA | 0.05 | NA | NA | NA | 0%- 22% |
| 26 | 468 | 606 | 77.20% | 496 | 606 | 81.80% | NA | NA | NA | <0.001 | 0.94 | NA | NA | 0.89-1.00 |
| 27 | 180 | 690 | 26.10% | 176 | 674 | 26.10% | NA | NA | NA | 0.99 | 1 | NA | NA | 0.78 - 1.27 |
| 28 | 176 | 470 | 37.40% | 178 | 470 | 37.90% | NA | NA | NA | 0.74 | 0.97 | NA | NA | 0.83 - 1.14 |
| 29 | 142 | 391 | 36.30% | 135 | 391 | 34.50% | NA | NA | NA | 0.65 | 1.05 | NA | NA | 0.87 - 1.27 |
| 30 | 525 | 1077 | 48.70% | 542 | 1080 | 50.20% | NA | NA | NA | 0.5 | 0.97 | NA | NA | 0.89 - 1.06 |
| 31 | 220 | 554 | 39.70% | 161 | 554 | 29.10% | NA | NA | NA | <0.001 | 1.37 | NA | NA | 1.16 - 1.61 |
| 32 | 76 | 280 | 27.10% | 100 | 284 | 35.20% | NA | NA | NA | 0.06 | 0.77 | NA | NA | 0.60 - 0.99 |
| 33 | 368 | 746 | 49.30% | 320 | 762 | 42.00% | NA | NA | NA | 0.004 | 1.17 | NA | NA | 1.05-1.31 |
| 34 | 34 | 107 | 31.80% | 8 | 85 | 9.40% | 9 | 67 | 13.40% | <0.001 | NA | NA | NA | −14.7-0.2 |
| 35 | 103 | 374 | 27.50% | 72 | 376 | 19.10% | NA | NA | NA | 0.007 | 1.44 | NA | NA | 1.10 - 1.87 |

Supplementary Table 2. Statistical Results of included studies

| Study ID | Randomization process | Deviations from intended interventions | Missing outcome data | Measurement of the outcome | Selection of the reported result | Overall Bias |
| --- | --- | --- | --- | --- | --- | --- |
| 1 | Low | Low | Low | Low | Low | Low |
| 2 | Low | Low | High | Low | Low | Some concerns |
| 3 | Low | Low | Low | Low | Low | Low |
| 4 | Low | Low | Low | Low | Some concerns | Low |
| 5 | Low | Low | Low | Low | Low | Low |
| 6 | Low | Low | Low | Low | Low | Low |
| 7 | Low | Low | Low | Low | Low | Low |
| 8 | Low | Low | Low | Low | Low | Low |
| 9 | Low | Low | Low | Low | Low | Low |
| 10 | Low | Low | Low | Low | Low | Low |
| 11 | Low | Low | Low | Low | Low | Low |
| 12 | Low | Low | Low | Low | Low | Low |
| 13 | Low | Low | Low | Low | Low | Low |
| 14 | Low | Low | Low | Low | Low | Low |
| 15 | Low | Low | Low | Low | Low | Low |
| 16 | Low | Low | Some concerns | Low | Low | Low |
| 17 | Low | Some concerns | Low | Low | Low | Low |
| 18 | Low | Low | Low | Low | Low | Low |
| 19 | Low | Low | Low | Low | Some concerns | Low |
| 20 | Low | Some concerns | Low | Low | Low | Low |
| 21 | Low | Low | Low | Low | Low | Low |
| 22 | Low | Low | Low | Low | Low | Low |
| 23 | Low | Low | Low | Low | Low | Low |
| 24 | Low | Low | Low | Low | Low | Low |
| 25 | Low | Low | Low | Low | Low | Low |
| 26 | Some concerns | Low | Low | Some concerns | Low | Some concerns |
| 27 | Low | Low | Low | Low | Low | Low |
| 28 | Low | Low | Low | Low | Low | Low |
| 29 | Low | Low | Low | Low | Low | Low |
| 30 | Low | Low | Low | Low | Low | Low |
| 31 | Low | Low | Low | Low | Low | Low |
| 32 | Low | Low | Low | Low | Low | Low |
| 33 | Low | Low | Low | Low | Low | Low |
| 34 | Low | Low | Some concerns | Low | Low | Low |
| 35 | Low | Low | Low | Low | Low | Low |

Supplementary Table 3. Detailed Judgments For Each Risk Of Bias Across All Included Studies

# Search Strategy

(((((Infertility[MeSH Terms])) OR ('Sterility, Reproductive'[Title/Abstract])) OR ('Reproductive Sterility'[Title/Abstract])) OR (Sterility[Title/Abstract])) OR (Subfertility[Title/Abstract])) OR (Sub-Fertility[Title/Abstract]))) OR (((((((((((((((((((((((((((("Fertility"[Mesh]) OR (Fecundability[Title/Abstract])) OR (Fecundity[Title/Abstract])) OR (Fertility Incentive[Title/Abstract])) OR (Fertility Incentives[Title/Abstract])) OR (Marital Fertility[Title/Abstract])) OR (Fertility, Marital[Title/Abstract])) OR (Natural Fertility[Title/Abstract])) OR (Fertility, Natural[Title/Abstract])) OR (World Fertility Survey[Title/Abstract])) OR (Fertility Surveys, World[Title/Abstract])) OR (Fertility Survey, World[Title/Abstract])) OR (Surveys, World Fertility[Title/Abstract])) OR (Survey, World Fertility[Title/Abstract])) OR (World Fertility Surveys[Title/Abstract])) OR (Fertility, Below Replacement[Title/Abstract])) OR (Below Replacement Fertility[Title/Abstract])) OR (Fertility Determinants[Title/Abstract])) OR (Determinant, Fertility[Title/Abstract])) OR (Determinants, Fertility[Title/Abstract])) OR (Fertility Determinant[Title/Abstract])) OR (Subfecundity[Title/Abstract])) OR (Differential Fertility[Title/Abstract])) OR (Fertility, Differential[Title/Abstract])) OR (Fertility Preferences[Title/Abstract])) OR (Fertility Preference[Title/Abstract])) OR (Preference, Fertility[Title/Abstract])) OR (Preferences, Fertility[Title/Abstract]))) OR (((((((((((((((((("Reproductive Techniques, Assisted"[Mesh]) OR (Assisted Reproductive Technique[Title/Abstract])) OR (Reproductive Technique, Assisted[Title/Abstract])) OR (Technique, Assisted Reproductive[Title/Abstract])) OR (Techniques, Assisted Reproductive[Title/Abstract])) OR (Assisted Reproductive Technics[Title/Abstract])) OR (Assisted Reproductive Technic[Title/Abstract])) OR (Reproductive Technic, Assisted[Title/Abstract])) OR (Reproductive Technics, Assisted[Title/Abstract])) OR (Technic, Assisted Reproductive[Title/Abstract])) OR (Technics, Assisted Reproductive[Title/Abstract])) OR (Assisted Reproductive Techniques[Title/Abstract])) OR (Reproductive Technology, Assisted[Title/Abstract])) OR (Assisted Reproductive Technologies[Title/Abstract])) OR (Assisted Reproductive Technology[Title/Abstract])) OR (Reproductive Technologies, Assisted[Title/Abstract])) OR (Technologies, Assisted Reproductive[Title/Abstract])) OR (Technology, Assisted Reproductive[Title/Abstract]))) AND (((((("JAMA"[Journal]) OR ("BMJ"[Journal])) OR ("Lancet"[Journal])) OR ("N Engl J Med"[Journal])))))) AND ((Randomized Controlled Trial))
